# On the Treatment of Whooping-Cough

**Published:** 1868-11

**Authors:** John C. Peters

**Affiliations:** New York


### ON THE TREATMENT OF WHOOPING-COUGH.

By JOHN C. PETERS, M.D., of New-York.

Is there any additional experience in the use of the decoction of chestnut leaves (castanea visca) in whooping-cough? Some time ago, Dr. Unzieker, of Cincinnati, gave it a fair trial in 30 cases, and felt satisfied that he had at last found a remedy to cope with this disease. It always gave decided relief in the first two weeks. First the cough was lessened and the patient rested easier at night, then the decline of all the symptoms became very rapid. He used three or four drachms of the leaves in boiling water, to be drank *ad libitum*, either hot or cold, and with or without sugar. Children do not dislike it.

The remedy is so simple, safe, and pleasant, that if this is not another instance of those tremendous exaggerations which so often make their way into the records of medicine, it should receive careful attention, especially as the remedy is so easily procured.

In the mild cases of whooping-cough, which generally last only two or three weeks, very little treatment is required. The suggestion of Dr. Snow, of Providence, to place small quantities of *carbolate of lime* in saucers about the room, where the child lives and sleeps, deserves attention as a prophylactic and disinfecting agent. It not only prevents others from contracting the disease, and destroys the contagion of the disorder on the spot, but also helps the patient. For Dr. Snow says, in all cases in which he tried it, a marked effect in diminishing the frequency and severity of the cough was quickly noticed.

The carbolate of lime is almost as cheap as the chloride, and need not be made unpleasant, for only sufficient should be put in the saucers to render its odor barely perceptible. Some should also be put in the spit-cups, slop-jars, etc., in which the children expectorate or vomit. For it is not only the breath of the child, but its secretions and excretions, which convey the disease to others. Thus Dr. Aitken says the disorder was first introduced into St. Helena by the captain of a ship, who sent the clothes of some children with whooping-cough on shore to be washed. We have known the disease to be conveyed several miles in the winter-time by the mother of a nursing infant. The child nursed just before its mother left the house; she at once put on her cloak and furs, and thus shut in the infection; then went to see her sister's children, whom she took on her lap and fondled. They contracted the disease at the usual time.

It is very probable, that there would be many more cases of *pertussis mitior* than *gravior*, if the hygienic management of the little sufferers were better attended to, than it generally is. Mild cases, which often last two or three weeks, may easily be converted into severe ones, by shutting the patient up in close rooms, or even heated houses, until these become filled with poisonous emanations, which probably unite with other effete matters and increase the malaria many fold. It is especially in these cases that change of air does so much good. The patient is benefited even by removal to so short a distance as half a mile; a sail across a river is also beneficial, although the distance be short, especially if the house be well ventilated while the patient is absent. This is all the mystery in those cases in which children recover from an almost hopeless state, even in a few hours after they have been sent away from home.

From the very commencement the child should be well clad, and kept in the open air as much as the weather will permit. Next to bad air, the most injurious agent is improper food, consisting of rich, sweet, indigestible, or greasy articles. These things not only irritate the stomach, but cause risings of acrid gases and fluids, which almost excoriate the pharynx and larynx and superinduce a stomach or throat-cough in addition to the specific disorder. And each aggravates the other. A light but nourishing diet of bread and milk, hominy, rice, corn-starch, farina, etc., with meat once a day, consisting of plain soup, the inside cuts of beef or mutton, chicken and fish without the skins; no fried potatoes, made gravies, or rich fricassees, etc., etc., will keep up the strength of the child and prevent all derangement of the stomach. Some bland or mucilaginous drink may be partaken of freely, like barley, or gum-Arabic water, flax-seed, or slippery-elm tea, etc. These will soothe and moisten the throat, and whole œsophageal and gastric mucous membrane.

It is not sufficiently known that in severe cases of whooping-cough, the stomach is singularly red and injected; and when it becomes inflamed, this is denoted not only by pain in the epigastrium, but also by the suppression of the glairy fluid, which should be thrown up by vomiting. In these cases, the little patient often lies in a state of complete exhaustion at the termination of the paroxysm of coughing, unable to discharge anything, either from the stomach or lungs, or even to whoop (AITKEN). Counter-irritation to the epigastrium, with or without aconite in soap—or weak hartshorn liniment to the back, are the most useful remedies in these cases. Even a few leeches to the stomach and a flax-seed poultice are not amiss. It is probably in these gastric cases, before they have advanced to far, that ipecac., has gained its great reputation.

The *first or catarrhal stage* of ordinary whooping-cough is like that of a feverish cold, and it is not until the fever remits and is about to pass away, that the cough which has previously distressed the patient, is followed by the characteristic whoop. The first stage may last from six or eight days to two or three weeks; but an experienced and observant physician will suspect the disease he has to deal with from the peculiar paroxysmal character of the coughs, which are divided by long intervals of comparative ease. Small doses of acetate of ammonia and ipecac., are useful in this stage: liquor ammoniæ acetatis ℥iss, syrupi ipecac., ℥ss. Dose: quarter, half, or whole teaspoonful in water several times a day, according to the age of the child; or ipecac., and spirits of nitre: spts. nit. dulce, ʒij; syrupi ipecac., ʒij; aq. cinnamomi ℥iss. Dose: quarter, half, or whole teaspoonful in a little water. Those who prefer it may give one or two drops of aconite; or two or three drops of digitalis; or one or two drops of gelsiminium, or quarter, half, or whole drops of tinct. of veratrum viride.

When the second or spasomodic stage sets in, the patient has a series of fits or paroxysms of severe coughing, occurring at irregular, but rather long intervals, and so rapid is the action of the diaphragm, that the air is almost instantly expelled from the lungs, and the patient half suffocated, turns black in the face. At length the crisis approaches, the diaphragm relaxes, and a violent inspiration follows, accompanied by the characteristic *whoop*. This sound remits, but soon returns, and thus convulsive inspiration and expiration continue, till the patient is at length relieved by a copious expectoration, or vomiting, or both. The matters expectorated are frequently thick, viscid, and muciform. Those vomited are thick and glairy, of much tenacity, semi-transparent, and often very copious (AITKEN). It is in this stage that alkalies are so very beneficial. Sub. carbonate of potash 20 grains; cochineal 10 grains; sugar, one-quarter oz.; water four oz.: rub together and strain. Dose: quarter teaspoonful four times a day for a child one year old; half a teaspoonful for one of two years; and a teaspoonful for one of four years. The cochineal is supposed by some to be an anti-spasmodic of some power, but it is more than probable that the alkali is the only efficient part of the prescription.

Dr. Hawley found the bromide of ammonium useful on account of its peculiar anæsthetic effect on the nerves of the larynx and pharynx. Dose: one or two grains thrice a day for infants; up to twelve grains for much older children.

The infusion of *wild thyme* slightly sweetened is said to effect great improvement of the spasmodic cough. Aitken thinks it is not very important which of the more decided sedatives or antispasmodics is selected, but thinks hyoscyamus and syrup of poppies are the safest and best. Still, there is no doubt that *conium malcolatum* is milder and better than hyoscyamus, belladonna, prussic acid, or opium. Potassæ sub. carb. gr. 20; aq. fœniculi three and a half ounces; tinct. conii maculat. half ounce. Dose: quarter, half, or whole teaspoonful, according to the age of the child. In severe cases: substitute 1 drachm of tinct. belladonna, for the half ounce of conium; or ten minims of dilute hydrocyanic acid.

Vollant and others prefer the powdered *root* of *belladonna* alone, giving one-fifth of a grain, first once, then twice, and finally four times a day, until the paroxysms begin to subside, which will certainly be on the third or fourth day. Then it is to be given at much longer intervals.

Others prefer *prussic acid* alone. Acid. hydrocyan. dilut. *m.* iv; syrupi simplicis, vel misturæ amygdalæ one drachm; aq. pur. seven drachms. Dose: half or one teaspoonful for a child nine months old. Or pulv. ipecac. compos. half to one and a half grain; ext. conii maculat. one gr.; pulv. cinnamomi gr. ij; sacch. alb. gr. iv; make a powder to be given at night, for children two years of age, when there is but little wheezing or vomiting, but a very troublesome night-cough.

The most frequent complication of this disease is acute *bronchitis*, when the mucus not only becomes deficient in quantity, but thick and viscid, teasing the patient with fruitless efforts to free it from the lungs, thus causing a frequent recurrence of the paroxysm. The alkaline treatment is the best here, with the addition of a few drops of aconite or digitalis. Or eqnal parts of aquæ laurocerasi, tinct. digitalis, and wine of antimony may be given in three, five, ten, or more drop doses.

When the bronchitis becomes more *chronic* and assumes the form of purulent inflammation, the pus secreted being formed into sputa and moderate in quantity, give: Misturæ ammoniaci one and a half ounce; aquæ cinnamomi, one and a half ounce; syrupi tolutani, half oz.; tinct. castorei, two drachms; tinct. opii camph., two drachms. Dose: one, two, or three teaspoonfuls, according to the age of the child, frequently (PARIS). When the pus is thrown up pure as from an abscess and in large quantities, astringents are required. Aluminis, gr. 24; acidi sulph. diluti *m.* 12; syrupi rheados (poppies) half an oz.; aq. pur. two and a half oz. Dose: one to three teaspoonfuls every six hours (WEST). Or: aluminis gr. 25; extracti conii maculat. gr. 12; syrupi rheados two drachms; aquæ anethi three oz. Dose: one or two teaspoonfuls every three or six hours, for a child two or three years old (GOLDING BIRD). Acidi nitrici diluti twelve drachms; tinct. cardamomi comp. three drachms; aquæ three and a half oz.; syrupi one oz. (GIBBS). One or two teaspoonfuls every two or three hours will cure in from two to fourteen days. Zinci sulphatis gr. eight; extracti belladonna, gr. two to five; aquæ four oz. Dose: one to four teaspoonfuls, two, four, or six times a day, for a child three years old.

If inflammation of the lungs set in, the treatment is nearly the same as for acute bronchitis. Or vini antimonii potassiotante, *m.* 30; vini ipecac., *m.* 10; tinct. aconit. rad. *m.* 5; tinct. camphoræ compos. *m.* 20; mucilag. acaciæ seven drachms. Dose: one or two teaspoonfuls every four hours, for a child four years of age (WEST).

But the most distressing accident is when the pleura becomes inflamed, for then the patient's sufferings are fearfully increased during the paroxysms of the cough. Then a roller bandage should be put on the chest to limit the movements of the ribs as much as possible. This may be moistened with an infusion of digitalis, or aconite, or opium; and the ordinary internal treatment well followed up. But the results of nineteen *post mortem* examinations made by Graily Hewitt during a recent epidemic, showed that the most frequent and fatal lesion of the lungs, in children from one month to four years of age, was collapse of the lung substance, or a return to the fœtal condition, or *atelectasis.* This calls for the free employment of stimulants and as strong liquid nourishment as can be digested. Tinct. nucis vomica, quarter, half, or one drop every few hours; or, tinct. cantharidis dr. j; tinct. camphoræ compos. ℥j; tinct. cinchonæ compos. dr. 10. Dose: quarter or half teaspoonful every two or four hours. Or three to five, or ten drops of aromatic spirits of ammonia.

In the third stage a number of remedies have been recommended. Assafœtida is much esteemed, and is considered by some physicians to be a specific not only in this, but in every other stage of the disease. Aitken says, it should be given in an emulsion, in the dose of one or two grains to a child two years old, repeated three or four times a day, or even as often as every two or three hours. Tinct. assafœtida dr. j; tinct. opii *m.* 10; ipecacuanhæ pulv. gr. 10; aquæ two oz. Dose: one teaspoonful every three or four hours (Reece). Assafœtidæ puræ dr. j; olei amygdalæ dulc. gutt. 20; rub together, and add mucilag. acaciæ two oz.; syrupi altheæ one oz. Dose: one teaspoonful every two hours for a child two years old (Kopp). Ext. lactucæ viros gr. iv; assafœtidæ depur. gr. viij; sacch. lact. vel. alb. gr. 40. Make eight powders; one every two or four hours.

Musk and castor have been largely used in severely spasmodic cases. Tinct. castorei half oz.; spts. lavend. compos. two dr.; aq. camphoræ ad. two oz. Dose: quarter or half teaspoonful in sweetened water. Ext. pulsatillæ gr. j; pulv. rad. valerian minor gr. three to six; elæosacchar, fœnicul, half drachm. Make twelve powders; one from two to four times a day. Herb. ledi palustris (wild rosemary) fol. sennæ dr. j; pulv. ipecac. gr. four; aquæ fervent four ozs.; sacch. alb. half to one oz.; liquor ammon. anisat. one drachm. Dose: one to four teaspoonfuls every two to four hours. Highly recommended by Buttner. Ext. dulcamaræ gr. 20; kali tartar depur gr. 40; aq. fœnicul one oz.; vini stibiat. half drachm. Dose: five to twenty drops in water, every two, four, or six hours.

The external treatment has received great attention. Olei succini dr. 2; liniment saponis compos. dr. 10; mix for a liniment similar to Roche's. Rub one drachm upon the back and chest, two or three times a day (Hooper). Spirit. camphoræ half oz.; tinct. opii. dr. 2; olei succini dr. 2; olei amygdalæ half oz. Mix for a liniment to be rubbed on the chest, night and morning (Savory).

Tanner prefers tinct. aconite dr. 2: soap liniment dr. 12; or a mixture of equal parts of tinct. belladonnæ, glycerine, and camphor liniment, to be rubbed on the back every night.

Such is the abundance of good and useful remedies, that all new suggestions should be received with great caution.—*Medical Gazette.*

